# Single Coronary Artery with Anomalous Rising of the Right Coronary Artery: A Rare Coronary Anomaly Diagnosed by 256-Multidetector Computed Tomography

**DOI:** 10.1155/2011/108709

**Published:** 2011-10-26

**Authors:** Gitsios Gitsioudis, Evangelos Giannitsis, Waldemar Hosch, Hans U. Kauczor, Hugo A. Katus, Grigorios Korosoglou

**Affiliations:** ^1^Department of Cardiology, University of Heidelberg, Im Neuenheimer Feld 410, 69120 Heidelberg, Germany; ^2^Department of Diagnostic and Interventional Radiology, University of Heidelberg, 69120 Heidelberg, Germany

## Abstract

Herein we report the diagnostic potential of cardiac computed tomography (cCT) to delineate the origin and course of an anomalous right coronary artery (RCA) originating from the midpart of the left anterior descended artery (LAD) in an adult with no other form of congenital heart disease. The patient was referred to our institution due to exertional dyspnea and suspected coronary artery disease. The patient underwent X-ray coronary angiography, and no high grade lesions were observed in the left coronary vessels. In the course of the mid-left-anterior-descending artery (LAD), an anomalous side branch coursing away from the left circumflex coronary artery (LCX) was observed, while a right coronary ostium could not be depicted. cCT confirmed the absence of a right coronary ostium, and the vessel originating from the mid LAD was identified as an anomalous RCA, which coursed anterior of the aorta and the pulmonary trunk.

## 1. Introduction

The incidence of isolated coronary anomalies is 0.3–0.9% and significantly higher (3–36%) in patients with other forms of congenital heart abnormalities [1–3]. Anomalies of an isolated right coronary artery (RCA) arising from the left coronary vessels are extremely rare with an incidence up to 0.035% [1]. The use of CT and MRI for the identification of such coronary artery anomalies is well established [4, 5].

Clinical relevance is mainly dependant on the anatomical course of the individual coronary anomaly which can vary between “within the range of normal” and sudden cardiac death, especially when additional atherosclerotic coronary disease is present. In particular, patients with coronary arteries originating from the opposite sinus with proximal vessel segments localized between the pulmonary trunk and the aorta, can present with syncope and sudden cardiac death and should therefore undergo bypass surgery [6, 7].

## 2. Case Presentation

**Figure 1:**
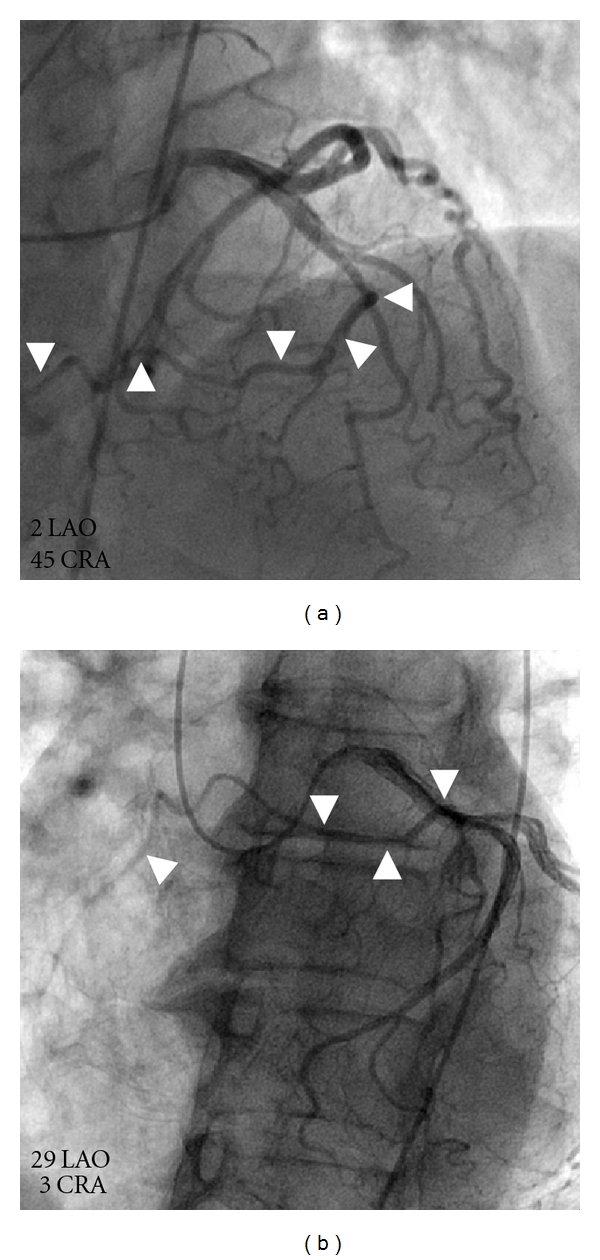
With conventional coronary angiography, an anomalous vessel originating from the mid-LAD was observed, coursing away for the circumflex coronary artery (white arrows in (a) and (b)). CRA indicates cranial; LAO: left anterior oblique.

**Figure 2:**
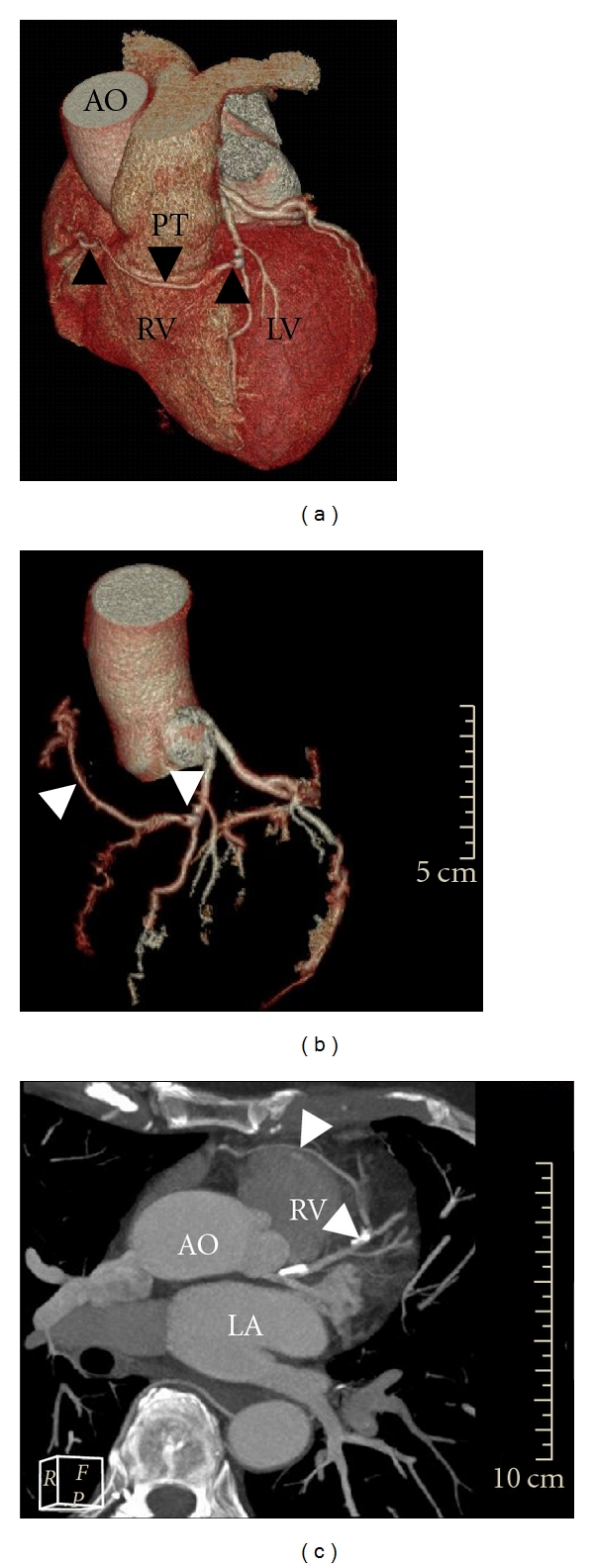
With coronary computed tomography, the presence of an anomalous RCA originating from the mid-LAD and coursing anterior of the aorta and the pulmonary trunk could be unambiguously illustrated. Volume rendering acquisitions (a)-(b) and multiplanar reconstructions (c) demonstrate the exact anatomical course of the anomalous vessel (black arrowheads in (a) and white arrowheads in (b) & (c)). (a) Overview of coronary vessel course in relation to the great arterial vessels aorta and pulmonary trunk; (b) coronary Tree; (c) axial maximum intensity projection. Ao indicates Aorta; LA: left atrium, RV: right ventricle, and PT: pulmonary trunk.

A 66-year-old woman with history of arterial hypertension was referred for evaluation of exertional dyspnea and atypical angina. An exercise of ECG yielded ST segment depression in leads V4–V6 indicating inducible myocardial ischemia. Echocardiography demonstrated normal left ventricular (LV) systolic function and concentric increased LV wall thickness (septal wall thickness of 11 mm). Coronary angiography exhibited mild coronary artery disease without high grade coronary lesions in the left coronary vessels. In the course of the left anterior descending artery (LAD) however, an anomalous side branch originating shortly after the first diagonal branch was depicted, which coursed away from the left circumflex coronary artery (LCX) (Figures 1(a) and  1(b)). Furthermore, during catheterization, a right coronary ostium could not be depicted neither by selective catheter intubation of the right coronary ostium nor by contrast injection in the aortic root. Therefore, 256-slice coronary computed tomographic angiogram (cCT) was performed in order to delineate the exact anatomical course of the anomalous branch and to determine the origin and course of the RCA. Cardiac CT scan was performed using a prospective scan protocol on a 256-slice Brilliance iCT scanner (Philips Best, Netherlands Healthcare) with a gantry rotation time of 270 ms, detector collimation of 2 × 128 × 0.625 mm^3^, with 256 overlapping slices of 0.625 mm thickness, and dynamic z-focal spot. For the cCT scan, 80 mL of contrast agent (Imeron 400, Bracco Imaging) and an effective dose of 3 mSv were applied. cCT showed moderate coronary calcification, and in agreement with invasive angiography, no high grade coronary lesions were observed. Furthermore, with CT images, the absence of a right coronary ostium was confirmed and the vessel originating from the mid-LAD was identified as an anomalous RCA, which indeed coursed anterior of the aorta and the pulmonary trunk to the inferior myocardial wall (Figures 2(a)–2(c)). Because the anomalous vessel exhibited such a “benign” course anterior to the great vessels, no increased risk for ischemia or sudden cardiac death was anticipated and the presence of LV hypertrophy was considered as the most probable explanation for the inducible ST depression observed during stress testing. Thus, the patient was continued on medical therapy including ß-blocker (100 mg of metoprolol) and angiotensin-converting enzyme inhibitor (10 mg of ramipril) medication for arterial hypertension, and aspirin (100 mg) and statin treatment (40 mg simvastatin) for mild coronary calcification, and CAD as diagnosed by cCT and invasive angiography.

## 3. Discussion

This case demonstrates the role of cardiac computed tomography for assessment of rare coronary anomalies in adults. The absence of an RCA ostium originating as a continuation of the proximal left anterior descending artery is extremely rare [1]. According to the Lipton classification of coronary anomalies, the presented case is of type LII (or LIIA) [1, 3, 8].

The use of cCT for the detection of coronary anomalies has been reported in a number of earlier studies [9–11]. The most common case of coronary anomalies is the separate origination of left anterior descending artery and circumflex artery from left sinus of valsalva, followed by the origin of the RCA from the left sinus valsalva, coursing between aorta and pulmonary artery. In contrast, single coronary artery with an anomalous right coronary vessel originating from the LAD is a very rare coronary artery anomaly with about 36 cases reported so far [12]. The incidence of CAD in these patients has been controversially discussed in the past [13, 14]. Halon et al. demonstrated that proximal points of branching may show significant intraluminal narrowing in selected patients with CAD. Our case showed calcification but no high grade coronary lesion at the origin of anomalous RCA. 

New onset angina, ECG changes, dyspnoea, syncope, and sudden cardiac death can be forms of presentation in adults and in the elderly [15, 16]. In this regard, lateral compression of the anomalous coronary artery may trigger myocardial ischemia causing exertional dyspnea, angina, or even severe arrhythmias resulting in sudden cardiac death. Intravascular ultrasound (IVUS) and X-ray coronary angiography represent the traditional methods for the assessment of coronary anomalies. In this regard, conventional X-ray angiography is the current clinical gold standard for the diagnosis of coronary anomalies and for the assessment of the presence and extent of concomitant CAD. Due to recent technical advances in cardiac magnetic resonance (CMR) and cCT in last years, these techniques are able to provide valuable incremental diagnostic information for the anatomical and functional diagnostic workup of such cases [9]. Particularly, the 3-dimentional nature and high spatial resolution of cCT allow for the unambiguous determination of the origin and proximal course of anomalous coronary vessels in relation to surrounding cardiovascular structures as the aorta and the pulmonary artery [10]. In addition, cardiac computer tomography can assess intraluminal narrowing, and if required, plaque characterization with high accuracy [17].

In conclusion, our case demonstrates the importance of coronary computed tomography for unambiguous delineation of both origin and vessel course in patients with very rare coronary anomalies.

##  Conflict of Interests

The authors declare that they have no competing interests with this paper.
